# High Prevalence of Severe Depression in Mexican Patients Diagnosed with HIV Treated with Efavirenz and Atazanavir: Clinical Follow-Up at Four Weeks and Analysis of TPH2 SNPs

**DOI:** 10.3390/jcm13247823

**Published:** 2024-12-21

**Authors:** Sandra Angélica Rojas-Osornio, Francisco Guerra-Castillo, Antonio Mata-Marín, Vladimir Paredes-Cervantes, Charmina Aguirre-Alvarado, Carolina Bekker-Méndez, Gilberto Pérez-Sánchez, José Molina-López, Mónica Ortiz-Maganda, Aurora Mercado-Méndez, Emiliano Tesoro-Cruz

**Affiliations:** 1 Escuela Superior de Medicina, Instituto Politécnico Nacional, Mexico City 11340, Mexico; sandii38@yahoo.com.mx; 2 Unidad de Investigación Biomédica en Inmunología e Infectología, Hospital de Infectología, “La Raza” National Medical Center, Instituto Mexicano del Seguro Social, Mexico City 02990, Mexico; pacoxguerra@gmail.com (F.G.-C.); charmina_burana@hotmail.com (C.A.-A.); bekkermendez@yahoo.com (C.B.-M.); moni_omaganda@yahoo.com.mx (M.O.-M.); 3 Infectious Diseases Department, Hospital de Infectología “La Raza” National Medical Center, Instituto Mexicano del Seguro Social, Mexico City 02990, Mexico; jamatamarin@gmail.com; 4 Laboratorio Central, Hospital de Especialidades “Dr. Antonio Fraga Mouret”, “La Raza” National Medical Center, Instituto Mexicano del Seguro Social, Mexico City 02990, Mexico; vlapace@hotmail.com; 5 Laboratorio de Psicoinmunología, Dirección de Investigaciones en Neurociencias del Instituto Nacional de Psiquiatría Ramón de la Fuente Muñiz, Mexico City 14370, Mexico; gilberto.perez.sanchez@inprf.gob.mx; 6 Unidad Periférica de Investigación Básica y Clínica en Enfermedades Infecciosas, Departamento de Salud Pública, División de Investigación, Facultad de Medicina, Universidad Nacional Autónoma de México, Mexico City 04510, Mexico; joseml@unam.mx; 7 Laboratorio de Patogenicidad Bacteriana, Unidad de Hemato-Oncología e Investigación, Hospital Infantil de México Federico Gómez, Facultad de Medicina, Universidad Nacional Autónoma de México, Mexico City 06720, Mexico; 8 Servicio de Higiene Mental del Hospital General, Centro Médico Nacional “La Raza”, Instituto Mexicano del Seguro Social, Mexico City 02990, Mexico; auroauroauro111@hotmail.com

**Keywords:** efavirenz (EFV), people with HIV (PWH), tryptophan hydroxylase type 2 (TPH2), depression, atazanavir/ritonavir (ATV/r)

## Abstract

Efavirenz (EFV) causes neuropsychiatric effects such as anxiety, depression, and suicidal thoughts in people with HIV (PWH). Depressive disorders have been associated with the Tryptophan hydroxylase type 2 (*TPH2*) gene. **Objectives**: This study determines the genotypes and allelic frequencies of three TPH2 single nucleotide polymorphisms (SNPs) in a Mexican cohort of HIV-1 treatment-naïve-patients and the severity of depressive symptoms at baseline and after a four-week clinical follow-up of antiretroviral treatment. **Methods**: In a pilot prospective study, eighty-one antiretroviral treatment-naïve patients were recruited from the Infectious Disease Hospital, National Medical Center “La Raza”, in Mexico City. Of these, 39 were treated using a set-dose combination regimen of tenofovir disoproxil fumarate/emtricitabine (TDF/FTC) plus EFV and 42 were treated with TDF/FTC plus atazanavir/ritonavir (ATV/r), and fifty-nine control volunteers. Genomic DNA was obtained from peripheral blood mononuclear cells. All DNA samples underwent qPCR utilizing TaqMan probes for the three *TPH2* SNPs studied. All participants underwent evaluation utilizing the Beck Depression Inventory. **Results:** Of the three SNPs examined, none exhibited any notable differences in the distribution of the alleles between the groups; nevertheless, rs4570625 TT and rs1386493 GG presented a twofold and fivefold greater risk of severe depression in PWH, respectively, independently of the treatment. Among PWH, those treated with EFV experienced severe depression at a higher rate of 90.4% after four weeks, compared to 87.5% in those treated with ATV/r. **Conclusions**: High rates of severe depression were identified in PWH, who presented the rs4570625 TT and rs1386493 GG polymorphic variants. Depression increased after four weeks of treatment and was higher with EFV than ATV/r. It is crucial to emphasize the necessity of conducting psychiatric monitoring for every patient with HIV and administering prompt antidepressant treatment.

## 1. Introduction

After the diagnosis of HIV, a process of assimilation and psychological coping begins that, depending on the personal and social resources of the patient, can lead to acute and serious problems and disorders such as depression, anxiety, and suicidal ideation; therefore, in addition to pharmacological treatment, HIV patients must be managed in a multidisciplinary manner, with special emphasis on their psychological health.

Depression is a complex biopsychosocial disease that is influenced by multiple factors that can trigger depressive episodes. Several reports have implicated that serotonin (5-HT) deficiency is associated with the pathogenesis of depression [1,2].

5-HT is an indolamine that is synthesized from tryptophan (an essential amino acid) by the enzymes tryptophan hydroxylase type 1 (TPH1) and tryptophan hydroxylase type 2 (TPH2) [3]. However, TPH2 is responsible for a higher proportion of 5-HT synthesis for brain function than *TPH1* [3,4]. Since TPH2 is the rate-limiting step for brain 5-HT biosynthesis, in a recent publication, we just showed to enhance enzymatic TPH2 activity within the raphe nucleus after in vivo transfection in mice following the ocular instillation of a plasmid (pIRES-hrGFP-1a-*TPH2*-FLAG), which contained the murine *TPH2* gene [5].

The TPH2 enzyme is brain-specific, and polymorphisms of this enzyme are associated with mood disorders.

A polymorphism is the presence of two or more variant forms of a specific DNA sequence that can occur between different people or populations. The most common type of polymorphism involves variation in a single nucleotide (also called single nucleotide polymorphisms, or SNPs). To identify a specific SNP, there is a reference SNP (rs) cluster ID (number), which is the nomenclature used for most SNPs.

Some of the most studied *TPH2* SNPs related to depression, suicide, and bipolar disorder are: (i) rs7305115 (associated with risk factors for suicide attempts and major depression) [6,7], (ii) rs1386493 (associated with suicide attempts) [8] and (iii) rs4570625 (associated with personality disorders, anxiety, depression, and major depressive disorders) [9,10,11].

Antiretroviral treatment (ART) has played an important role in the control of HIV-1 infection and has enabled a longer quality of life for people with HIV (PWH). International HIV treatment guidelines recommend two nucleoside reverse transcriptase inhibitors plus a non-nucleoside reverse transcriptase inhibitor (NNRTI), boosted protease inhibitor (PI) or integrase inhibitor as first-line ART to achieve adequate RNA HIV suppression [12]; however, in most cases, ART may cause neuropsychiatric adverse effects (NPAE’s).

Efavirenz (EFV) is an NNRTI that is commonly used in combination with lamivudine/zidovudine, abacavir/lamivudine, and tenofovir/emtricitabine [13]. It is the most extensively utilized NNRTI due to its efficacy and excellent pharmacokinetics [14,15,16]. Nevertheless, its use is currently dwindling in many countries because of reported NPAEs [14,16], which could be explained in part by the CYP2B6 polymorphisms of some populations that provide the variation in general pharmacokinetics that trigger a high plasma concentration or a longer plasma half-life of EFV [17,18,19,20,21,22,23,24,25,26,27].

Another reason why EFV causes neuropsychiatric alterations could be its influence on the *TPH2* gene. Recently, we reported dysregulation in the *TPH2* gene after the administration of oral chronic EFV within the brainstem, hypothalamus, and amygdala and depression-related processes in behavior trials in mice [28].

The use of EFV in Mexico has declined since December 2019; however, it is used even in a few regions in Mexico and Central America.

In 2020, the Mexican Institute of Social Security (IMSS) introduced a new drug regimen, Biktarvy^®^ (bictegravir/emtricitabine/tenofovir alafenamide), for the treatment of PWH. This approach is groundbreaking, as it has a lower likelihood of causing viral resistance and fewer side effects [29]. On the other hand, Atazanavir (ATV), a PI, can be administered with a pharmacokinetic enhancer such as ritonavir or cobicistat and is always used in combination with other medications such as tenofovir/emtricitabine. Among the adverse effects reported for this drug are changes in heart rhythm, severe skin rash, allergic reactions, liver conditions, and drug interactions, but there are no reports focusing on depression [30].

The current study aims to analyze the genotypes and allele frequencies of *TPH2* SNPs (rs7305115, rs4570625, and rs1386493) in a Mexican cohort that included eighty-one PWH and a control group of fifty-nine volunteers. Additionally, the study aimed to determine the severity of depressive symptoms at baseline and after a four-week clinical follow-up of antiretroviral treatment with a set-dose combination regimen of tenofovir disoproxil fumarate/emtricitabine (TDF/FTC) plus EFV and TDF/FTC plus Atazanavir/ritonavir (ATV/r).

## 2. Materials and Methods

### 2.1. Study Design and Subjects

A pilot prospective cohort study was conducted from January 2017 to December 2018 at the Immunology, Infectology Research Unit, and Infectious Diseases Department of the Intectology Hospital “Dr. Daniel Méndez Hernández”, of National Medical Center “La Raza”, Instituto Mexicano del Seguro Social (IMSS), located in Mexico City, México.

This study was conducted in accordance with the Helsinki Declaration guidelines and approved by the Bioethical Committee of the IMSS National Research (CNIC-R-2016-785-033). Eighty-one ART-naïve HIV-1-positive patients were recruited from the Infectious Diseases Department, meeting the protocol’s selection criteria.

The patients received comprehensive care from an infectious disease physician, psychiatrist, and nutritionist, which involved updating vaccines and undergoing a workshop on maintaining medication compliance. Subsequently, each patient completed the Beck’s Depression Inventory (BDI) and signed an informed consent form before commencing either TDF/FTC plus EFV (39 patients) or TDF/FTC plus ATV/r (42 patients) treatments. Some PWH were treated with EFV or ATV/r because they were randomly assigned to start a regimen according to the national and international guidelines at that time. Randomization was performed using the digital system (MEDSHARING: randomizer for clinical trial) to one of the two arms made up of (a) EFV (600 mg once daily) + TDF/FTC (300/200 mg once daily) or (b) ATV/r (300 mg/ 100 mg once daily) + TDF/FTC (300/200 mg once daily). The label design was open-ended, so subjects and researchers were aware of group assignments.

Demographic characteristics, such as sex, age, and psychoactive substances, were obtained through a validated questionnaire administered at the time of blood collection. To preserve confidentiality, data were processed using unique identifiers. The BDI survey was performed during the first medical appointment.

Fifty-nine healthy subjects were included as a control group. Upon agreement to participate and subsequent signature of the written informed consent form by all involved participants, BDI was applied, and a blood sample was collected.

### 2.2. Blood Sampling

Five milliliters of peripheral blood were collected from both the PWH and control volunteers using a standardized procedure. Blood samples were drawn using ethylenediaminetetraacetic acid Vacutainer tubes (BD, San Jose, CA, USA) and stored at room temperature for subsequent analyses. Four milliliters of serum were also taken from PWH to learn their biochemical parameters and the changes they could present due to the drugs.

### 2.3. Isolation of Peripheral Blood Mononuclear Cells (PBMCs)

PBMCs were isolated using Ficoll-Hypaque density gradient centrifugation (Sigma-Aldrich, Darmstadt, Germany). Five milliliters of whole blood were collected and diluted 1:1 with sterile phosphate buffer solution (PBS). The samples were then placed in 15 mL tubes on a Ficoll-Hypaque gradient and separated by centrifugation (Eppendorf^®^, Model 5810R, Merck KGaA, Darmstadt, Germany) at room temperature and 850 g for 20 min using an oscillating rotor with the brake disconnected. The PBMCs were collected from the interface and washed twice in PBS by centrifugation at 2500 rpm for 5 min each time. The resulting cell pellet was resuspended in 0.2 mL of PBS and stored in a cryogenic box at −80 °C until required.

### 2.4. Genotyping

Genomic DNA was extracted from the PBMCs pellet using the QIAamp DNA Mini Kit (Valencia, CA, USA), according to the product specifications. The DNA obtained was quantified using NanoDrop 2000 equipment (Thermo Scientific^®^, Alcobendas, Madrid, España) and stored in a cryogenic box at −80 °C until used. Three SNPs, rs7305115, rs4570625, and rs1386493, were genotyped using TaqMan^®^ SNP Genotyping Assays (Foster City, CA, USA). Allelic discrimination analysis was performed using an ABI 7500 Fast System real-time PCR instrument (Life Technologies, Foster City, CA, USA). Genotyping was carried out with a 25 μL reaction mixture, consisting of 20 ng of genomic DNA, 1.2 μL of 20X assay (Applied Biosystems, Foster City, CA, USA), 12.5 μL of Genotyping Master Mix (Applied Biosystems, Foster City, CA, USA), and ddH_2_O up to a final volume of 25 μL. The genomic DNA was denatured by heating at 95 °C for 10 min and underwent 40 cycles at 95 °C for 15 s and 60 °C for 60 s, extension for 40 s at 72 °C, denaturing for 40 s at 9 °C, and then the final extension for 6 min at 72 °C. All genotyping was blindly performed without knowledge of the samples’ clinical status or background data.

### 2.5. Statistical Procedures

All participants, including PWH and control subjects, underwent depression assessments using the BDI [31,32]. Its purpose was to identify the presence of depression and evaluate its severity. The BDI is a straightforward, self-administered questionnaire consisting of 21 items and four categories: no depression (0–12 points), mild depression (13–20 points), moderate depression (21–25 points), and severe depression (>26 points).

Statistical analyses were performed using GraphPad Prism 6.0 statistical software (Inc., La Jolla, CA, USA).

Hardy–Weinberg equilibrium (HWE) analysis was performed using the X^2^ test. The distribution of allele frequencies and genotypes for all *TPH2* polymorphisms was determined using the allele counting method. The genetic models assessed comprised differences in allelic frequency, recessive and dominant models, overdominant models, homozygote contrasts, and heterozygote contrasts. These models were calculated with odds ratios (ORs) associated with 95% confidence intervals for each SNP. For demographic data, frequencies, percentages, means, and standard deviations were used, and the X^2^ test was used to compare proportions between groups using the BDI. Fisher’s exact test was used to compare the groups in relation to the occurrence of depression in those carrying polymorphisms in each of the study groups. Statistical significance was determined for *p*-values less than 0.05.

## 3. Results

Eighty-one treatment-naïve males (100%) aged 19 to 42 years (mean age 28.0 ± 8.3 years) with HIV and fifty-nine control volunteers (30.5% men and 69.4% women, mean age 28.2 ± 11.3 years) were recruited. Table 1 displays the sociodemographic and biochemical characteristics of the study groups. As reported in previous studies [30,33,34,35,36], lipid levels (triglycerides) were altered in the EFV-treated group, while the ATV/r-treated group showed increases in triglycerides and unconjugated bilirubin (refer to Table 1).

### 3.1. TPH2 Genotyping Analysis

All SNPs showed genotype frequencies consistent with HWE proportions.

The frequencies and distributions of alleles among the *TPH2* SNPs studied in the case–control association analysis was determined, including 81 cases and 59 controls.

Table 2 displays all frequencies and distributions of alleles among the analyzed SNPs.

We included three SNPs in our analysis, which did not show statistically significant differences in the distribution of the rs7305115, rs4570625, and rs1386493 between cases and controls.

PWH from the DF/FTC + EFV group had a frequency of the SNP rs7305115, with GG in 11 cases (28.2%), and of rs4570625, with TT in 6 cases (15.3%) and of the rs1386493, with GG predominating in 25 cases (64.1%). The TDF/FTC + ATV/r group had the presence of the SNP rs7305115, with GG in 13 cases (30.9%), the rs4570625 with TT in 5 cases (11.9%), and rs1386493 with GG predominating in 28 cases (66.6%).

The controls showed a frequency of SNP rs7305115 GG, *n* = 15 (25.4%), rs4570625 TT, *n* = 8 (13.5%) and predominating rs1386493 GG, *n* = 39 (66.1%).

#### 3.1.1. Beck Depression Inventory (BDI)

**Figure 1:**
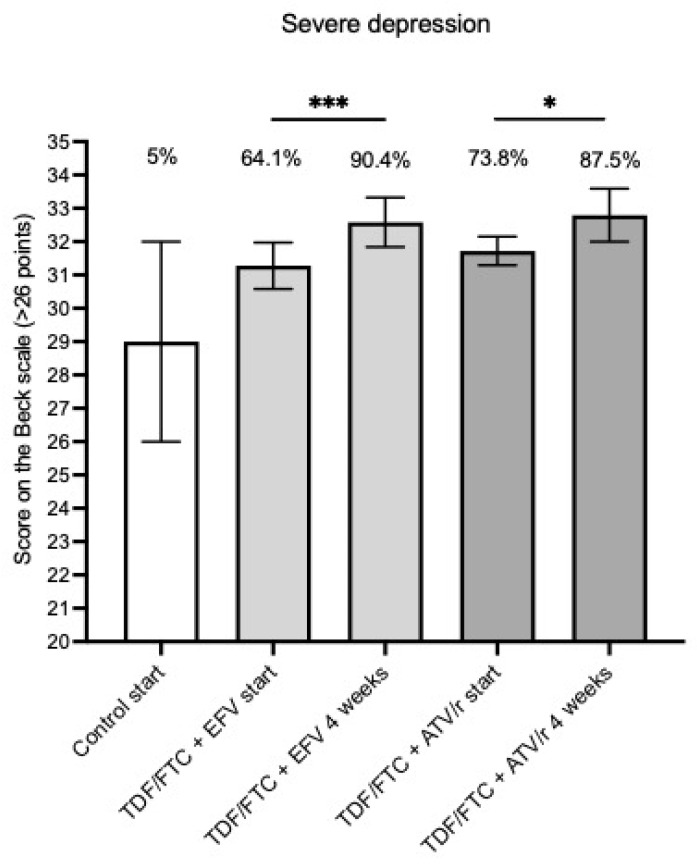
Graphic representation of the differences in severe depression between groups. Graphic representation differences in severe depression (>26 points) presented by the BDI study individuals at the beginning and 4 weeks later. An increase can be observed in both treated groups, with greater significance in patients treated with TDF/FTC + EFV (*** *p* < 0.0001) compared to patients treated with TDF/FTC + ATV/r (* *p* < 0.05).

All cases and controls underwent BDI evaluation. The initial score from PWH treated with TDF/FTC + EFV showed that 64.1% had severe depression, which rose to 90.4% after 4 weeks of treatment. On the other hand, the initial BDI score of PWH treated with TDF/FTC + ATV/r showed that 73.8% had severe depression, which increased to 87.5% after 4 weeks. The number of patients with severe depression increased with both treatments, but it was more evident in those who were treated with EFV (EFV X^2^ = 19.085; df: 1; *** *p* < 0.0001; ATV/r X^2^ = 5.383; df: 1; * *p* = 0.0203). In the control group, severe depression was observed in 5% of volunteers (Table 3 and Figure 1).

#### 3.1.2. Relationship Between Polymorphisms and Depression

**Figure 2:**
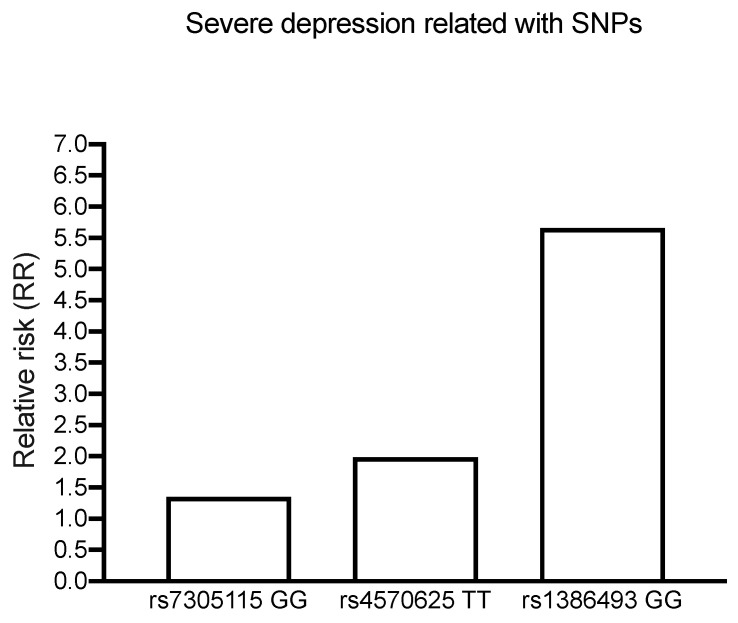
Graphic representation of the relative risk (RR) between the presence of SNPs and severe depression. Regardless of the group (control, TDF/FTC + EFV or TDF/FTC +ATV/r), the three SNPs studied showed a greater risk of severe depression: rs7305115 presented a 1.5-fold greater risk, rs4570625 polymorphic variant presented a twofold greater risk, while those with the rs1386493 polymorphic variant presented more than fivefold greater risk.

According to the results obtained between polymorphisms and depression association, we observed in PWH a twofold greater risk of severe depression among individuals with the rs4570625 TT polymorphic variant and a fivefold greater risk of severe depression among those with the rs1386493 GG polymorphic variant regardless of the treatment administered (Fisher exact test rs4570625 TT: EFV, RR = 1.8; *p* = 0.211; ATV/r, RR = 2.1; *p* = 0.124; rs1386493 GG: EFV, RR = 5.5; *** *p* < 0.0001; ATV/r, RR = 5.81; *** *p* < 0.0001) (Table 4 and Figure 2).

## 4. Discussion

In this study, we found a high prevalence of severe depression in individuals with an HIV diagnosis. The proportion of individuals with severe depression increased following four weeks of treatment, and a higher rate was observed in those receiving TDF/FTC + EFV compared to those receiving TDF/FTC + ATV/r.

However, social factors might influence depression in PWH. Moreover, PWH have a higher risk of depression, anxiety, insomnia, and suicide. Even prior to the initiation of antiretroviral treatment, this is associated with the uncertainty of the disease, ignorance and family impact, so they have a higher frequency of neuropsychiatric disease, including depressive problems.

Factors significantly associated with depression among HIV-positive patients on antiretroviral therapy included employment status [AOR  =  0.22 (95% CI 0.13–0.36)], the patient’s highest CD4 count [AOR  =  6.99 (95% CI 2.81–17.38)], duration of months on antiretroviral therapy [AOR  =  5.05 (95% CI 2.38–10.74)], and the presence of chronic non-communicable diseases [AOR  =  7.90 (95% CI 4.21–14.85)]. The highest proportion of HIV-positive patients taking antiretroviral drugs exhibited depression. Employment was identified as a preventive factor, whereas having a low CD4 count, recently initiating antiretroviral therapy, and having chronic non-communicable diseases were associated with increased odds of depression among HIV-positive patients on antiretroviral therapy [37].

Some individuals may be more susceptible to depression due to biological factors; it is estimated that genetics account for about one-third of depression risk, while environmental factors account for two-thirds [38].

In previous studies, analysis of the *TPH2* gene has revealed variants associated with depression, suicide, and bipolar affective disorder. In fact, there is information reporting that SNP rs7305115 is associated with risk factors, including suicide attempts and major depression. Three independent studies have reported this association of patients with major depressive disorder with and without suicidal behavior and suggest that this polymorphism may be a predictive factor for suicidal behavior [6,7,39].

The SNP rs1386493 decreases the efficiency of normal *TPH2* RNA splicing, giving rise to a truncated protein (TPH2-TR) by alternative splicing. *TPH2*-TR lacks enzymatic activity and negatively affects the dominant function of long-lasting *TPH2*, which reduces 5-HT production. The mRNA formed for *TPH2*-TR is present in post-mortem brain tissue [38].

Another polymorphism that has been linked to several psychiatric and/or behavioral phenomena, as well as suicidal intent behavior, is SNP rs4570625 [40]. In a meta-analysis study, they reported that this SNP has a strong association with major depression, and according to the number of articles analyzed, epidemiological credibility persists; however, more studies are required to provide strong evidence for other weak associations [10]. Other reports on functional studies describe the impact of the SNP rs4570625 on amygdala responses to emotional stimuli [41]. In another study, Yong-Ku Kim et al. detected an association between rs4570625 and panic disorder [42].

In this study, we observed that the risk of severe depression increases when the individual presents the polymorphisms rs4570625 TT and rs1386493 GG. For PWH, being aware of their diagnosis is one of the most significant risk factors for the development of depression. The emotional response to the condition is often intense, resulting in psychological difficulties, depression, psychosis, or mania. However, it is important to also take into account environmental factors such as the family and social environments, diet, sports, and recreational activities.

On the other hand, the sociodemographic characteristics of PWH showed that 53.8% with TDF/FTC + EFV and 41.4% with TDF/FTC + ATV/r regimen are smokers and 58.9% and 63.4%, respectively, are alcohol drinkers (Table 1). Depression and alcoholism are linked to significant morbidity, disability, and mortality. Several epidemiological studies [43,44,45,46] have demonstrated the extent of comorbidity between these disorders. In 2003, Sher et al. compared depressed patients with and without a previous history of alcoholism and found that depressed subjects with a history of alcoholism had lower cerebrospinal fluid monoamine metabolites [47]. Compared to depressed subjects without an alcoholism history, those with such a history had decreased cerebrospinal fluid homovanillic acid (HVA) levels, which resulted in a decrease in dopamine availability. The alcohol intake restored dopamine levels, and these individuals were prone to smoking tobacco, aggression, and suicidal ideation.

Furthermore, the dopaminergic system in patients with comorbid depression and alcohol dependence is likely to be more impaired than in patients with each diagnosis alone. Moreover, individuals with a history of alcoholism exhibit additional biological abnormalities, including alterations in the ɤ-aminobutyric acid, N-methyl-D-aspartate, endogenous opioid, and serotonergic systems in the brain [44,45,46]. In certain studies conducted with alcohol-dependent patients, the results showed a higher incidence of the rs1386493 GG *TPH2* gene [8,48,49]. In our study, we observed that more than half of the PWH (64.2%) presented the SNP rs1386493 GG, a risk factor for alcohol-related issues according to previous reports [8,48,50], which could have clinical significance in terms of depression alcohol-related development among PWH.

Researchers have extensively studied the *TPH2* gene in relation to psychiatric morbidity. Several trials have investigated various *TPH2* polymorphic variants, with rs4570625 showing the strongest association with neurological disorders [10,40,41,42]. Regarding our results about the rs4570625 TT *TPH2* gene in PWH, we found that 15.3 and 11.9% from TDF/FTC + EFV group EFV and TDF/FTC + ATVr, respectively, carried this SNP. This SNP may be a biological factor and a potential risk factor for emotional regulation disorders like anxiety, depression, and aggression, as well as higher amygdala activity in response to emotional stimuli [51].

In this study, a high prevalence of severe depression was found among HIV-diagnosed patients, presenting a twofold greater risk of severe depression regardless of the treatment administered. The prevalence increases after four weeks of treatment with TDF/FTC + EFV and TDF/FTC + ATV/r, with a higher prevalence in patients treated with TDF/FTC + EFV compared to those treated with TDF/FTC + ATV/r.

According to several reports previously mentioned in this study [15,17], EFV is related to the development of depression in some individuals, but there are no reports of ATV/r; hence, it is necessary to explore the relationship between ATV/r and depression with a larger cohort of patients due to this drug is currently used.

The focus of this study was limited to the high prevalence of severe depression found among male HIV-diagnosed patients. Here, we only considered patients from the men’s clinic; therefore, women with HIV were not considered.

Although the impact of *TPH2* SNPs in terms of depression in PWH was evaluated, showing a higher risk of severe depression in carriers of the rs4570625 TT and rs1386493 GG polymorphic variants, the sample was small, and the cohort will need to be expanded to clarify this result.

Unfortunately, genetic factors affecting the pharmacokinetics of EFV are not included in this study, nor were the CYP2B6 polymorphisms determined, which are important in terms of general pharmacokinetics and trigger a high plasma concentration or longer plasma half-life of EFV. However, it is an area of opportunity that can be studied in the future with a larger cohort of patients. Furthermore, we could not make other associations because we lacked information on the cases of alcoholism and smoking from the control group, which is considered a limitation of this study. Regarding the BDI used in this trial, we recognize it should be complemented with other questionnaires such as the HADS-D, PHQ-9, Hamilton Depression Scale, or the Montgomery–Asberg Rating Scale.

Future research may include genotyping, which could supplement a personalized therapeutic strategy alongside measuring plasma EFV concentrations, enhancing the safety and tolerance of this medication. Furthermore, it is crucial to evaluate female PWH receiving treatment with these drugs to analyze the degree of depression according to their cyclic hormonal variations that could interfere with their emotional state. There are no experimental clinical studies that have evaluated this gender difference, so it could be interesting for future studies to carry out this type of approach.

## 5. Conclusions

High rates of severe depression were identified in PWH, who presented the rs4570625 TT and rs1386493 GG *TPH2* polymorphic variants. Depression increased after four weeks of treatment, which was higher for EFV than ATV/r. Furthermore, we considered the implications of these findings to be important for health policy and HIV treatment, so it is crucial to emphasize the necessity of conducting psychiatric monitoring for every patient with HIV and administering prompt antidepressant treatment when it is required.

## Figures and Tables

**Table 1:** Sociodemographic and biochemical characteristics of the study groups.

		Control *n* (%)	TDF/FTC + EFV *n* (%)		TDF/FTC + ATV/r *n* (%)	
**Anthropometry**						
Sex	Male	18 (30.5)	39 (100)		42 (100)	
	Female	41 (69.4)	0		0	
Age (years)	19–29	40 (67.7)	30 (76.9)		23 (54.7)	
	30–39	2 (3.3)	8 (20.5)		16 (38)	
	40–49	17 (28.8)	1 (2.5)		3 (7.1)	
BMI	Underweight		2 (6)		1 (2.8)	
	Normoweight		22 (57.5)		25 (60)	
	Overweight		11 (27.2)		12 (28.5)	
	Obesity		4 (9)		4 (8.5)	
**Psychoactive Substances**						
Smoker			21 (53.8)		17 (41.4)	
Alcohol drinker			23 (58.9)		27 (63.4)	
**Laboratory Elements**						
			Start	After 4 weeks	Start	After 4 weeks
Cr mg/dL (0.6–1.2)			0.91 (± 0.2)	0.90 (± 0.1)	0.88 (± 0.16)	0.94 (± 0.13)
ALT U/L (4–36)			32.13 (± 18.9)	50.77 (± 54.0)	38.26 (± 24.2)	32.5 (± 21.48)
AST U/L (8–33)			25.6 (± 9.5)	36.27 (± 25.8)	29.85 (± 17.4)	24.42 (± 7.1)
TC mg/dL (200)			139.94 (± 36.2)	163.09 (± 33.36)	133.15 (± 46.0)	147.4 (± 32.9)
TG mg/dL (150)			151.4 (± 106.8)	***191.18 (± 103.0)***	138.82 (± 67.3)	***199.8 (± 111)***
TBIL mg/dL (0.1–1.2)			0.73 (± 0.3)	0.42 (± 0.16)	0.67 (± 0.31)	***3.34 (± 1.98)***
UBIL mg/dL (0.8)			0.45 (± 0.2)	0.25 (± 0.11)	0.41 (± 0.27)	***3.38 (± 3.52)***

In terms of biochemical characteristics, the panel of laboratory parameters shows changes in lipid levels (triglycerides) in both treated groups and increases in total and unconjugated bilirubin in the ATV/r-treated group (shown in italics and bold numbers). BMI—body mass index; Cr—creatinine; ALT—alanine aminotransferase; AST—aspartate aminotransferase; TC—total cholesterol; TG—triglycerides; TBIL—total bilirubin; UBIL—unconjugated bilirubin.

**Table 2:** Frequency of single nucleotide polymorphisms among the study groups.

Polymorphism	Genotype	Control *n* (%)	TDF/FTC + EFV *n* (%)	OR (95% CI)	*p* Value	TDF/FTC + ATV/r n (%)	OR (95% CI)	*p* Value
rs7305115 A > G	A/A	12 (20.3)	8 (20.5)	0.989	0.983	12 (28.5)	0.638	0.338
	A/G	32 (54.2)	20 (51.2)	1.126	0.774	17 (40.4)	1.743	0.172
	G/G	15 (25.4)	11 (28.2)	0.868	0.760	13 (30.9)	0.760	0.540
rs4570625 G > T	G/G	21 (35.5)	14 (35.8)	0.987	0.975	12 (28.5)	1.382	0.458
	G/T	30 (50.8)	19 (48.7)	1.089	0.836	25 (59.5)	0.703	0.388
	T/T	8 (13.5)	6 (15.3)	0.862	0.800	5 (11.9)	1.161	0.806
rs1386493 A > G	A/A	3 (5.0)	3 (7.6)	0.643	0.598	3 (7.1)	0.696	0.666
	A/G	17 (28.8)	11 (28.2)	1.030	0.948	11 (26.1)	1.141	0.771
	G/G	39 (66.1)	25 (64.1)	1.092	0.838	28 (66.6)	0.975	0.952

For all single nucleotide polymorphisms evaluated, we observed genotype frequencies in the HWE proportion. It displays all frequencies and distributions of alleles among the three analyzed SNPs, which did not show statistically significant differences in the distribution of the rs7305115, rs4570625, and rs1386493 between cases and controls. TDF/FTC (Tenofovir alafenamide/Emtricitabine); EFV (Efavirenz); ATV/r (Atazanavir/ritonavir); OR (Odds ratio); CI (Confidence interval).

**Table 3:** Beck Depression Inventory (BDI).

Beck Depression Inventory (BDI)	Control *n* = 59 (%)	Patients on TDF/FTC + EFV *n* = 39 (%)		Patients on TDF/FTC + ATV/r *n* = 42 (%)	
		Start	After 4 Weeks	Start	After 4 Weeks
No depression	44 (74.5)	0 (0)	0 (0)	0 (0)	0 (0)
Mild depression	8 (13.5)	0 (0)	0 (0)	0 (0)	0 (0)
Moderate depression	4 (6.7)	14 (35.8)	4 (9.5)	11 (26.1)	5 (12.5)
Severe depression	3 (5.0)	25 (64.1)	35 (90.4) ***	31 (73.8)	37 (87.5) *

BDI score: The BDI score was statistically higher in the EFV-treated group than in the ATV/r-treated group after 4 weeks of treatment. EFV: X^2^ = 19.085; df = 1; *** *p* < 0.0001; ATV/r: X^2^ = 5.383; df = 1; * *p* = 0.0203. No depression = 0–12 points, mild depression = 13–20 points, moderate depression = 21–25 points, and severe depression = >26 points.

**Table 4:** Relationship between depression and presence of polymorphisms.

BDI Basal Score							
	Control *n* (%)	TDF/FTC + EFV *n* (%)	RR	*p* Value	TDF/FTC + ATV/r *n* (%)	RR	*p* Value
SNP	rs7305115GG						
No depression	12 (20.3)	0 (0)	0.55	** 0.002	0 (0)	0.53	** 0.001
Mild depression	2 (3.3)	0 (0)	0.59	0.515	0 (0)	0.58	0.509
Moderate depression	0 (0)	0 (0)	0.00	---	0 (0)	0.00	---
Severe depression	0 (0)	8 (20.5)	+ infinity	*** 0.0004	12 (28.5)	+ infinity	**** <0.0001
SNP	rs4570625TT						
No depression	4 (6.7)	0 (0)	0.59	0.148	0 (0)	0.57	0.138
Mild depression	2 (3.3)	0 (0)	0.59	0.515	0 (0)	0.58	0.509
Moderate depression	0 (0)	1 (2.5)	+ infinity	0.398	0 (0)	+ infinity	>0.9
Severe depression	2 (3.3)	4 (10.2)	1.85	0.211	5 (11.9)	2.12	0.124
SNP	rs1386493GG						
No depression	23 (38.9)	0 (0)	0.48	**** <0.0001	0 (0)	0.46	**** <0.0001
Mild depression	5 (8.4)	0 (0)	0.58	0.153	0 (0)	0.56	0.073
Moderate depression	1 (1.6)	1 (2.5)	1.20	>0.9	0 (0)	0.58	>0.9
Severe depression	4 (6.7)	24 (61.5)	5.50	**** <0.0001	26 (61.9)	5.81	**** <0.0001

Fisher’s exact test was used to compare the groups in relation to the occurrence of depression in the basal score of BDI. It was observed that PWH with the rs4570625 polymorphic variant presented a twofold greater risk of severe depression, while those with the rs1386493 polymorphic variant presented a fivefold greater risk of severe depression regardless of the treatment administered. SNP—single nucleotide polymorphism. RR—Relative risk. (** *p* < 0.01), (*** *p* < 0.001), (**** *p* < 0.0001).

## Data Availability

Data are unavailable due to privacy or ethical restrictions.
